# Adaptive ERK signalling activation in response to therapy and in silico prognostic evaluation of EGFR-MAPK in HNSCC

**DOI:** 10.1038/s41416-020-0892-9

**Published:** 2020-05-19

**Authors:** Chao Rong, Marie F. Muller, Fang Xiang, Alexandra Jensen, Wilko Weichert, Gerald Major, Peter K. Plinkert, Jochen Hess, Annette Affolter

**Affiliations:** 1 Department of Pathology, School of Biology & Basic Medical Sciences, Soochow University, Suzhou, China; 2 Department of Otorhinolaryngology, Head and Neck Surgery, Heidelberg University Hospital, Heidelberg, Germany; 3 Department of Otorhinolaryngology, Wuhan Center Hospital, Tongji Medical College, Huazhong University of Science and Technology, Wuhan, China; 4 Department of Radiation Oncology, University Hospital Giessen, Giessen, Germany; 5 Institute of Pathology, Technical University Munich, Munich, Germany; 6 Department of Radiation Oncology and Radiation Therapy, Heidelberg University Hospital, Heidelberg, Germany; 7 Research Group Molecular Mechanisms of Head and Neck Tumors, German Cancer Research Center (DKFZ), Heidelberg, Germany; 8 Department of Otorhinolaryngology, Head and Neck Surgery, University Hospital Mannheim, Medical Faculty Mannheim of Heidelberg University, Mannheim, Germany

**Keywords:** Head and neck cancer, Prognostic markers, Molecular medicine, Head and neck cancer, Prognostic markers

## Abstract

**Background:**

Head and neck squamous cell carcinoma (HNSCC) patients frequently develop treatment resistance to cetuximab, a monoclonal antibody against EGFR, as well as radiotherapy. Here we addressed extracellular signal-regulated kinase 1/2 (ERK1/2) regulation by cetuximab or fractionated irradiation (IR) and conducted in silico prognostic evaluation of the EGFR-MAPK axis in HNSCC.

**Methods:**

Expression of ERK1/2 phosphorylation (pERK1/2) was determined in HNSCC cell lines, which were treated with cetuximab or fractionated-IR. Furthermore, the effect of fractionated IR on pERK1/2 was confirmed in an ex vivo HNSCC tissue culture model. Expression and prognostic significance of EGFR-ERK axis was evaluated in a cohort of radiotherapy plus cetuximab-treated HNSCC. Correlations among EGFR-MAPK signalling components and association between transcript and protein expression profiles and patient survival in HNSCC were analysed using publicly available databases.

**Results:**

ERK1/2 phosphorylation was rebounded by prolonged cetuximab administration and was induced by fractionated IR, which could be suppressed by a MEK inhibitor as a radiosensitiser. In silico assessments suggested that EGFR-MAPK cascade genes and proteins could predict HNSCC patients’ survival as a prognostic signature.

**Conclusions:**

Activation of ERK1/2 signalling contributes to the cellular defence of HNSCC against cetuximab and fractionated IR treatment. EGFR-MAPK axis has a prognostic significance in HNSCC.

## Background

The epidermal growth factor receptor (EGFR) and its ligands promote the malignant behaviour of different tumours by cellular processes such as proliferation, differentiation, antiapoptotic signalling, angiogenesis, and metastasis.^1^ EGFR is overexpressed in up to 90% of head and neck squamous cell carcinomas (HNSCC), associated with an unfavourable prognosis.^2^ By EGFR phosphorylation intracellular signalling cascades, for example, Ras/Raf/MAPK (mitogen-initiated protein kinase) and PI3K/AKT (phosphatidylinositol 3-kinase) are activated, which mediate cellular proliferation and survival.^3,4^

Systemic therapy has been the pillar for treating patients with recurrent/metastatic (R/M) HNSCC. Historically, the therapeutic regimen comprised a platinum-based chemo doublet regimen with either cisplatin or carboplatin and 5-fluorouracil. Vermorken et al. proposed the superiority of incorporating cetuximab, a monoclonal antibody (mAb) targeting EGFR, in combination with cisplatin/carboplatin and fluorouracil in patients with R/M HNSCC.^5^ Multiple clinical studies have confirmed a survival advantage for patients with locally advanced HNSCC, who were treated with cetuximab in combination with radiotherapy as compared with irradiation alone considering progression-free survival (PFS) and disease-free survival (DSS).^6^ Nevertheless, the response and sensitivity of the individual patient to cetuximab vary significantly, and it is known that EGFR inhibitors have limited effects as a monotherapy.^5,7^ Numerous patients are refractory to anti-EGFR treatment, which points out to the fact that EGFR expression is not the only determining factor in treatment response. It has been shown that the MAPK-ERK signalling pathway contributes to the lack of response to EGFR inhibition.^8,9^ ERK activation by oncogenic effectors such as HRAS^10^ or MET^11^ has been postulated to trigger cetuximab resistance.

Resistance against radiotherapy is a limiting factor for the curative treatment of HNSCC. Exposure to ionising radiation (IR) is supposed to cause compensatory activation of intracellular signalling cascades securing tumour cell survival. There is evidence of complex functional linkages between the formation mechanisms of radioresistance and the IR-induced signalling pathway MEK/ERK. This cascade is known as “survival pathway” and its activation has anti-apoptotic and proliferation-enhancing effects. Previous studies have already demonstrated IR-dependent induction of MEK/ERK in established HNSCC cell lines and an ex vivo tissue culture model.^4,12,13^ Targeting ERK1/2 kinase provides potential therapeutic opportunities for a broad spectrum of cancers.^14^ Therapy response is impacted by a pronounced intra-tumorigenic heterogeneity in HNSCC. The selection of radioresistant tumour cell sub-clones after fractionated radiotherapy as clinically applied is thereby facilitated. However, many previous studies made statements on molecular mechanisms in radioresistance after single dose irradiation considerably exceeding a dosage of 2 Gy.^15–17^ The clonal selection of radioresistant tumour cells under fractionated irradiation is at this moment not taken into account.

Main objectives of the study were to define the ERK1/2 regulation by cetuximab or fractionated IR and to investigate the potential of the clinically tested MEK inhibitor PD-325901 (PD-901) as a radiosensitiser. Moreover, correlations among EGFR-MAPK signalling components and association between transcript and protein expression profiles and patient survival in HNSCC were analysed using publicly available databases.

## Methods

### Cell culture

Human HNSCC cell lines FaDu, Cal27, SCC4, SCC9 and SCC25 were purchased from ATCC (https://www.lgcstandards-atcc.org). Cells were maintained in Dulbecco’s Modified Eagle’s Medium (DMEM) supplemented with 10% foetal bovine serum (Invitrogen, Germany), 2 mM l-glutamine and 50 μg/ml penicillin-streptomycin in humidified and sterile conditions with 6% CO_2_ at 37 °C. Cell cultures were regularly screened to exclude mycoplasma contamination (Venor®GeM Classic Mycoplasma Detection Kit, Minerva Biolabs) according to manufacturer’s recommendation, and the authentication of all cell lines was confirmed by the Multiplex Human Cell Line Authentication Test (Multiplexion, Germany, latest update April 2019).

### Reagents and antibodies

The EGFR inhibitor cetuximab (Merck KGaA) was dissolved in sodium chloride 0.9%. The MEK inhibitor PD-0325901 (PD-901) (SIGMA-Aldrich, Inc.) was dissolved in dimethyl sulfoxide (DMSO) and stored in aliquots, in accord with the manufacturers’ instructions. Details and concentrations were summarised in the specific experiments. All information on the antibodies used was listed in Supplementary Table S1.

### Western blot analysis and Immunofluorescence (IF) staining

Cells were lysed by ice-cold RIPA buffer containing proteasome-inhibitor cocktail and phosphatase-inhibitor cocktail (Sigma-Aldrich, Germany). The lysates were centrifuged at 4 °C and the supernatant was collected. Protein concentration of RIPA lysates was measured utilising BCA protein assay kit (Thermo Scientific, Germany).^18^ A volume of homogenate containing 20 μg of total protein was separated by 10% SDS‐polyacrylamide gel electrophoresis (PAGE). Gels were transferred to nitrocellulose membranes and blotted using primary antibodies and secondary antibodies. Membranes were incubated in enhanced chemiluminescence solution (Thermo Scientific, Germany) and measured with ImageQuant LAS500 system (GE Healthcare, Germany). Immunofluorescence staining was done as described elsewhere. Cells were grown on sterile coverslips and fixed with phosphate-buffered saline (PBS)—4% paraformaldehyde for 15 min at room temperature. After being washed with PBS, the cells were permeabilised with 0.5% Triton X-100 buffer in PBS and blocked with PBS-1 % bovine serum albumin-0.2% Tween 20 for 30 min at room temperature. Coverslips were incubated with phospho-ERK1/2 (CST9101, Cell Signaling Technology, Germany) antibody in blocking buffer (PBS-1% bovine serum albumin-0.2% Tween 20) for overnight at 4 °C. Cell nuclei were visualised by incubation of cells with Hoechst 33342 (Calbiochem Merck, Germany) and cytoskeleton rearrangement was visualised by incubation with anti-Cy3 (Dianova, Germany). Finally, the coverslips were mounted with Mowiol. Image acquisition was made by fluorescence microscopy (Leica DMLB microscope) using a digital camera (Nikon digital camera DXM1200) and the Nikon Act-1 software.

### Colony formation assay

To investigate the clonal expansion of HNSCC cells upon fractionated irradiation with or without PD-901, 300 or 1000 HNSCC cells were seeded per well in six-well plates and irradiated on four consecutive days with a daily dose of 2 grey (Gy) using X-RAD 320 (Precision X-Ray, North Branford, CT USA), or kept untreated as controls. Half of the irradiated cells were administrated daily with PD-901 at the indicated concentrations. To assess the sensitivities of tumour cells upon cetuximab, 1000 or 3000 HNSCC cells were seeded per well in six-well plates and half of the cells were treated every second day with cetuximab at the indicated concentrations. After 10–14 days in culture, cell clones were visualised by crystal violet staining, and the total number of colonies was counted as described in ref. ^19^. The survival fraction was calculated using a freely available software Clono-counter according to ref. ^20^

### Ex vivo culture

Fresh tissue HNSCC samples (*n* = 3, one from the oropharynx, one from the oral cavity, one from the larynx) were procured immediately after surgical resection at the Department of Otorhinolaryngology, Head and Neck Surgery, Heidelberg University Hospital, Germany. Informed consent was obtained from all patients after the review of the local ethics board (ethic vote S-396/2012. Samples were processed as previously described.^4^ For ex vivo analysis of tumour response to fractionated irradiation, tumour sections were maintained in six-well plates with inserts (Thinsert, Greiner Bio-One, Frickenhausen, Germany) in Dulbecco modified Eagle medium, supplemented with 10% foetal bovine serum and antibiotics (penicillin 100 U/mL and streptomycin 100 μg/mL). After 1 day in culture, samples were irradiated with an intensity of 2 Gy on 4 consecutive days. Non-treated controls were processed in parallel. The medium was changed every second day. The tissue slices were harvested 72 h posttreatment to be evaluated for histopathological and immunohistochemical (IHC) features.

### HNSCC patient samples

HNSCC samples were derived from a cohort, which has previously been characterised in more detail.^21,22^ Paraffin-embedded tumour samples were obtained from the tissue bank of the National Center for Tumor Diseases (NCT) Heidelberg and the Institute of Pathology, University Hospital Heidelberg, Germany. The tissue samples were used in accordance with the ethics committee of the Heidelberg Medical Faculty (ethic vote S206/2005). All subjects gave written informed consent by the Declaration of Helsinki. The treatment concept consisted of a cetuximab (ErbituxVR) loading dose of 400 mg/m^2^ body surface followed by weekly administrations of 250 mg/m^2^ body surface for the duration of the radiotherapy. Irradiation was applied at a dosage of 66–72 Gy (primary tumour and involved lymph nodes) and 54–57.6 Gy (bilateral neck), respectively. The final analysis was based on 22 patients with HNSCC (Supplementary Table S2). Clinical and therapeutic follow-up of the cohort was assessed retrospectively.

### Tissue microarray and IHC

Tissue microarrays (TMAs) preparation and IHC staining were described previously.^23–25^ Immunostaining was visualised with DAB peroxidase substrate (Vector Laboratories, Burlingame, USA) according to the manufacturer’s instructions. Detailed information on antibodies for IHC was listed in Supplementary Table S1. Counterstaining was done by haematoxylin to visualise tissue integrity. Stained TMAs were scanned using the Nanozoomer HT Scan System (Hamamatsu Photonics, Japan) and were evaluated by three independent observers using the NDP Viewer software (version 1.1.27). The evaluation considered the relative amount of positive cancer cells and the staining intensity. Both values were multiplied to calculate the final immunoreactivity score (IRS). Expression patterns of each protein were defined as high and low expressing subgroups.

### In silico prognostic evaluation of EGFR-MAPK in HNSCC

TCGA-HNSCC (*n* = 502) and TCPA-HNSCC (*n* = 328) datasets were downloaded from the cBio Cancer Genomics Portal (http://cbioportal.org) and The Cancer Proteome Atlas (https://tcpaportal.org) in December 2018, respectively. Data sets from two independent cohorts have been described in detail elsewhere.^26,27^ Correlation analyses of EGFR-MAPK signalling components were performed by using R-project *corrplot*. The prognostic evaluation of EGFR-MAPK signalling components in HNSCC patients was performed by *SurvExpress* web tool.^28^ A heat map representation of the protein expression values was made by supervised clustering with *ClustVis*.^29^

### Statistics

SPSS 22 for Windows (SPSS Inc., USA) and GraphPad Prism version 8 (GraphPad Software, USA) were used for statistical analysis. In vitro data were represented as mean values + SEM of three independent experiments. The patients were divided into two risk groups by estimating the prognostic index (PI) and a Cox model algorithm as described previously.^28^ The differences between groups were analysed by Student’s *t* test. Correlation analysis was performed by Spearman’s rho. Overall survival (OS) was calculated using the Kaplan–Meier method and compared by the Log-Rank test. *p*-values < 0.05 were considered as statistically significant.

## Results

### Regain of ERK phosphorylation upon prolonged cetuximab treatment in HNSCC cell lines

**Fig. 1:**
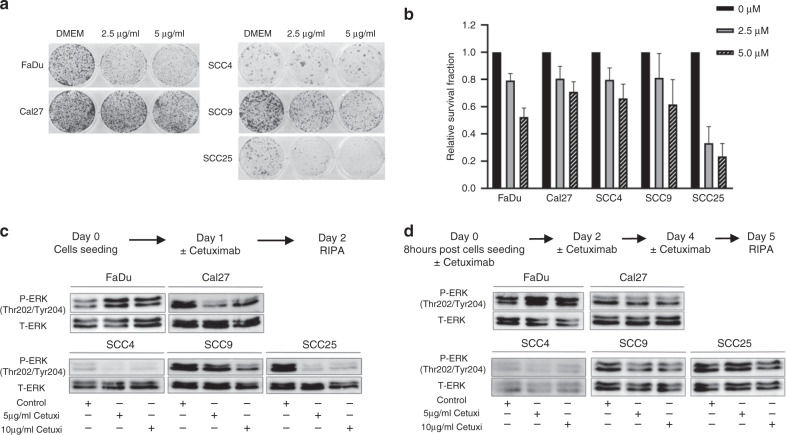
Regain of ERK phosphorylation upon prolonged cetuximab treatment in HNSCC cell lines. **a** Representative staining of a CFA with control (DMEM) or cetuximab-treated HNSCC cell lines. **b** Graphs represent the relative survival fraction of HNSCC cell lines after treatment with indicated concentrations of cetuximab. DMEM-treated control cells were normalised to one and bars represent mean values + SEM of three independent experiments. Expression of pERK1/2 and total ERK1/2 protein in HNSCC cell lines after a single dose (**c**) and prolonged (**d**) cetuximab treatment was determined by western blot analysis with whole cell lysates. Schematic protocols of cetuximab treatment were summarised.

CFAs were performed to address the impact of cetuximab on survival and clonal expansion of several HNSCC cell lines. Cell lines displayed a heterogeneous response to EGFR blockage and SCC25 were identified as the most sensitive cell line to cetuximab treatment among all cell lines tested (Fig. 1a, b). High sensitivity of SCC25 cells towards cetuximab was accompanied by a strong decrease in ERK1/2 phosphorylation upon a single dose treatment. A less pronounced and in part concentration-dependent reduction of ERK1/2 phosphorylation by cetuximab was also detected for Cal27 and SCC9 cells (Fig. 1c). By contrast, FaDu cells showed elevated pERK1/2 levels in response to a single dose of cetuximab (Fig. 1c). In terms of prolonged cetuximab treatment, a similar trend with minor differences in ERK1/2 phosphorylation was observed for FaDu and SCC9 cells (Fig. 1d). Furthermore, there was no sustained pERK1/2 inhibition in Cal27, SCC4, and SCC25 after repeated cetuximab treatment, which all showed reduced pERK1/2 levels after short-term treatment. Collectively, these data demonstrate that most HNSCC cell lines adapt to prolonged cetuximab treatment concerning ERK1/2 phosphorylation.

### Induced ERK phosphorylation upon fractionated irradiation is eliminated by MEK inhibition

**Fig. 2:**
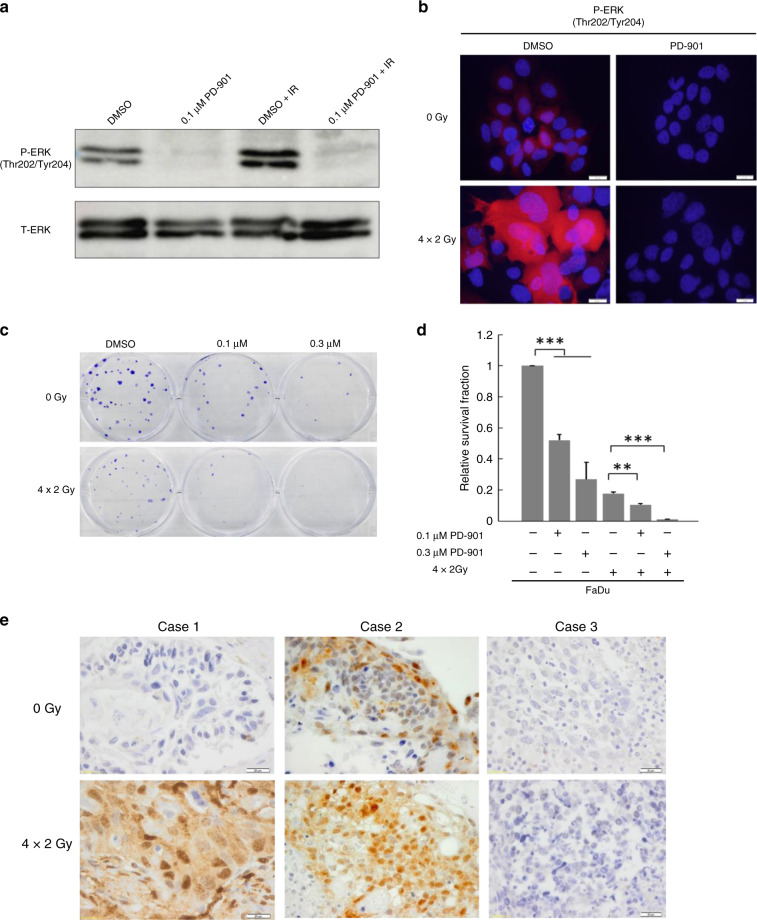
Fractionated irradiation (IR) -induced ERK signalling can be eliminated by MEK inhibitor. pERK1/2 protein levels in FaDu cells after fractionated IR or (and) PD-901 treatment were determined by immunofluorescence (IF) staining (**a**) and western blot analysis with whole cell lysate (**b**). Cell nuclei were counterstained with Hoechst H33342 (blue signal). Scale bars = 20 μm. **c** Representative staining of control (DMSO) or PD-901 treated FaDu with or without fractionated IR. **d** Graphs indicate the relative survival fraction of FaDu cells after treatment with given concentrations of PD-901 with or without fractionated IR. DMSO-treated control cells are normalised to one and bars depict mean values + SEM of three independent experiments. *p < 0.05, **p < 0.005, ***p < 0.0005. **e** Representative IHC staining of pERK1/2 in ex vivo tumour tissues with or without fractionated IR (4 × 2 Gy).

We previously reported post-radiogenic activation of the MAPK pathway in HNSCC by a single dose irradiation.^4^ To confirm our findings in vitro, FaDu cells were treated with the fractionated-IR scheme and ERK1/2 phosphorylation after fractionated IR was determined by western blot analysis (Fig. 2a), which have been previously characterised by strong activation of pERK1/2 after irradiation with a single dose.^4^ In line with previous results, fractionated IR revealed a distinct upregulation of ERK1/2 phosphorylation, which was particularly strong in a subpopulation of surviving tumour cells as demonstrated by IF staining (Fig. 2b). Basal and IR-induced ERK1/2 phosphorylation were abolished by application of the specific MEK inhibitor PD-901 (Fig. 2a, b). These results indicate that post-radiogenic activation of MAPK signalling most likely contributes to cellular defence in response to treatment and can be tackled by MEK inhibition. To investigate whether MEK inhibitor treatment alters the radiosensitivity of FaDu cells, CFAs were performed after combined treatment with fractionated IR and PD-901. PD-901 mono-treatment revealed a concentration-dependent reduction in the survival fraction (Fig. 2c, d). Moreover, administration of PD-901 sensitised FaDu cells to fractionated IR in a concentration-dependent manner (Fig. 2d). The impact of PD-901 in combination with cetuximab and fractionated irradiation on FaDu and Cal27 was also assessed by CFAs, which showed that administration of the MEK inhibitor strongly facilitated the treatment efficacy of cetuximab and (or) fractionated irradiation in HNSCC cell lines (Supplementary Fig. S1). Taken together, these findings indicate that the MEK inhibitor PD-901 acts synergistically with radiotherapy and (or) cetuximab treatment impairing clonogenic survival. To refine these results and to adapt to a clinical setting, we investigated the impact of fractionated IR with a daily dose of 2 Gy on ERK1/2 phosphorylation in three independent ex vivo tumour cultures. Two samples displayed elevated ERK1/2 phosphorylation as compared to controls (Fig. 2e). The third ex vivo tumour culture showed negative basal ERK1/2 phosphorylation and fractionated IR caused no induction, reflecting the distinct heterogeneity in HNSCC.

### Expression and prognostic significance of EGFR-ERK axis in radiotherapy plus cetuximab-treated HNSCC

**Fig. 3:**
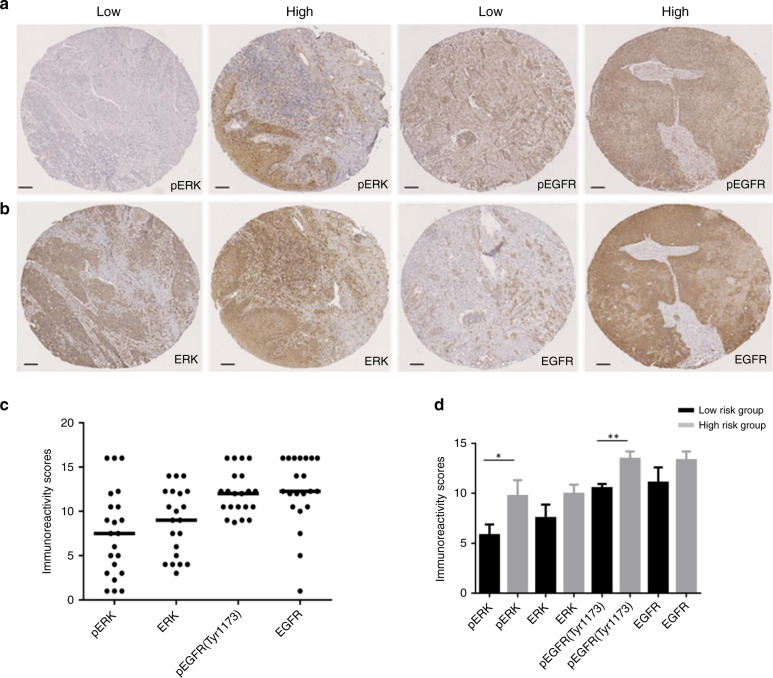
Tissue microarray-based prognostic evaluation of pERK1/2, ERK1/2, pEGFR and EGFR in HNSCC. Representative pictures of IHC stained primary tumour sections for pERK1/2 and pEGFR (**a**) with the low and high Immunoreactivity scores (IRS), the corresponding ERK1/2 and EGFR staining were also presented (**b**). Counterstaining of cell nuclei was performed with haematoxylin (signal in blue) to demonstrate tissue architecture. Scale bars equal 100 µm. IRS of the four proteins are summarised (**c**) and compared between risk groups (**d**). Bars depict mean values + SEM of IRS. *p < 0.05, **p < 0.005.

TMAs were available for IHC staining revealing informative data for pERK1/2 (*n* = 22) and for pEGFR(Tyr1173) (*n* = 21) (Fig. 3a, b). IHC staining revealed a heterogeneous staining pattern for pERK1/2, ranging from undetectable to prominent staining intensity (IRS 1–16) (Fig. 3c). pEGFR (Tyr1173) was expressed with a higher staining intensity and with a higher number of positive cells in tumour specimens, compared to pERK1/2 (Fig. 3c). Matching total ERK and total EGFR expression levels served as controls. To plot the performance of potential prognostic biomarkers pERK1/2 and pEGFR(Tyr1173), we divided the samples by the ordered median of the prognostic index (PI) to designate low- and high-risk groups, whereas the two groups are not significantly different in overall and progression-free survival as well as clinical features due to the small cohorts. The PI is the linear component of the exponential function in the Cox model.^28^ Both expression levels of pERK1/2 and pEGFR(Tyr1173) were significantly elevated in the high-risk groups as compared to the low-risk groups (Fig. 3d). There is no significant difference between the high and low-risk group on ERK1/2 and EGFR expression. These data indicated that high expression of pERK1/2 and pEGFR(Tyr1173) could serve as risk factors for unfavourable clinical outcome of HNSCC patients.

### EGFR-MEK-ERK gene signature is predictive for survival of HNSCC patients

**Fig. 4:**
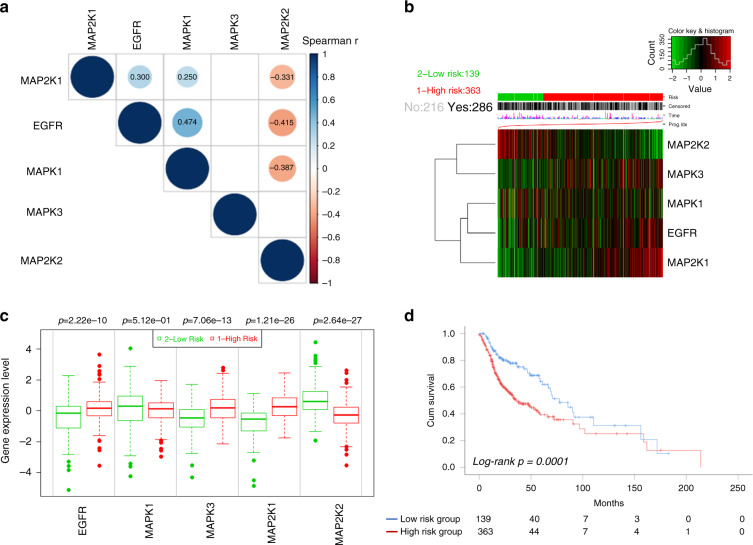
Prognostic analysis of EGFR-MEK-ERK gene signature in HNSCC by TCGA database. **a** Visualisation of a correlation matrix for EGFR-MEK-ERK pathway gene expression values. Positive correlations were shown in blue and negative correlations in red. Only correlation *p*-values < 0.01 were displayed and the detailed correlation coefficients were indicated. **b** Heatmap represented EGFR-MEK-ERK pathway gene expression values (rows) and tumour samples (columns) in different risk groups. **c** Box and whisker plots of EGFR-MEK-ERK pathway gene expression values were compared between risk groups by Student’s *t* test. **d** Overall survival of risk groups was evaluated by Kaplan–Meier survival plot and Log-Rank test. Total number of patients at risk were displayed at indicated time points.

To further evaluate the prognostic value of the EGFR-MEK-ERK pathway, the related gene expression profiles were analysed with the TCGA-HNC dataset comprising 502 patients. First, we investigated whether transcriptional levels of key nodes in EGFR-MEK-ERK signalling correlated with each other in HNSCC. Spearman’s correlation analyses were performed among EGFR, MAP2K1, MAP2K2, MAPK1, MAPK3 transcript levels and revealed a strongly positive correlation between EGFR and MAPK1 or MAP2K1. Interestingly, a significantly negative correlation was found between MAP2K2 and EGFR as well as MAPK1 transcript levels (Fig. 4a). Next, a heat map based on EGFR, MAP2K1, MAP2K2, MAPK1, MAPK3 transcript levels were generated by the *SurvExpress* web tool and two clusters with an either low or high prognostic risk were defined by PI and Cox fitting (Fig. 4b). With the exception of MAPK1, differential expression of all other genes was highly significant between the high-risk as compared to the low-risk group (Fig. 4c). As expected, MAP2K2 transcript levels were downregulated in the high-risk groups. Furthermore, Kaplan–Meier plot and Log-Rank analysis revealed a significant difference in overall survival between the high and low risk group (Fig. 4d; Hazard ratio [HR] = 1.91, confidence interval [CI] = 1.37–2.64). This finding was confirmed in the two independent cohorts (Fig. S1). In summary, these findings demonstrated that a gene expression signature related to the EGFR-MEK-ERK pathway predicts the clinical outcome for HNSCC patients.

### The predictive value of EGFR-MEK-ERK gene signature for survival is confirmed on protein level

**Fig. 5:**
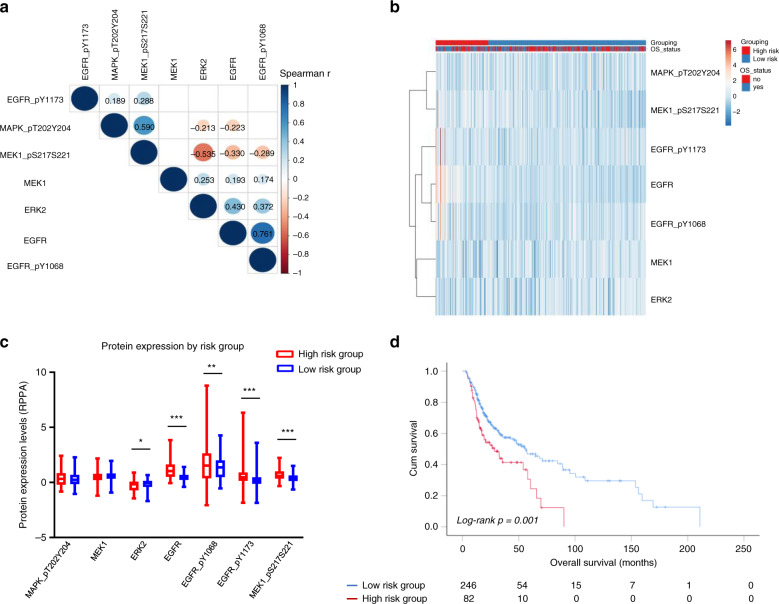
Prognostic analysis of EGFR-MEK-ERK proteins expression in HNSCC by TCGA database. **a** Visualisation of a correlation matrix for EGFR-MEK-ERK pathway protein expression values. Positive correlations were shown in blue and negative correlations in red. Only correlation *p*-values < 0.01 were displayed and the detailed correlation coefficients were indicated. **b** Heatmap represented EGFR-MEK-ERK pathway protein expression values (rows) and tumour samples (columns) in different risk groups by supervised clustering. **c** Box and whisker plots of EGFR-MEK-ERK pathway protein expression values were compared between risk groups by *t* test. **d** Overall survival of risk groups was evaluated by Kaplan–Meier survival plot and Log-Rank test. Total number of patients at risk were displayed at indicated time points.

To confirm the prognostic value of the gene signature related to EGFR-MEK-ERK pathway on the protein level, we analysed the proteomic dataset from TCPA-HNSCC. Spearman’s correlation analysis was applied to evaluate the association among EGFR-MEK-ERK signalling components, including EGFR, pEGFR(Tyr1068), pEGFR(Tyr1173), MEK1, pMEK1, ERK2, pERK1/2. Our data show that pEGFR(Tyr1173) is positively correlated with levels of MEK and ERK phosphorylation, while phospho-EGFR(Tyr1068) revealed a strong positive correlation with EGFR, MEK1, ERK2 protein levels, and a significantly negative relationship with levels of MEK phosphorylation (Fig. 5a). Heatmap clusters indicated different groups with low or high prognostic risk, as described previously (Fig. 5b). Differential protein expression analysis confirmed our TMA data that pEGFR(Tyr1173) were significantly elevated in the high-risk groups as compared to the low-risk groups. In addition, EGFR, pEGFR(Tyr1068), pMEK1 were significantly induced in the high-risk groups as compared to the risk groups (Fig. 5c). Kaplan–Meier survival analysis of HNSCC cohort based on protein expression has validated the findings from the genomic dataset, which suggests EGFR-MEK-ERK cascade proteins could predict HNSCC patient survival as a prognostic signature (Fig. 5d).

## Discussion

Cetuximab is the only targeted therapy that has been proven effective for the treatment of HNSCC in both locally advanced (LA) and R/M settings. The EXTREME regimen (cetuximab combined with cisplatin and 5-FU) has remained the standard of care for the first-line treatment of R/M-HNSCC.^30,31^ A combination of cetuximab with RT has been proven to be superior compared to RT alone in a Phase 3 trial for locally advanced HNSCC (LA-HNSCC).^32^ However, concomitant cetuximab was not compared with RCT in a Phase 3 study yet, and the use of cetuximab has been restricted to patients who are considered unfit for a platinum-based RCT.

Furthermore, the lack of prognosticators of response to cetuximab has restricted a widespread use. Further novel small molecule inhibitors and monoclonal antibodies did not achieve a favourable outcome in HNSCC patients so far. It seems likely that cancer cells that develop adaptive response and resistance against therapy use their capacity to upregulate survival pathways such as MAPK-ERK signalling. This process might facilitate the expansion of tumour clones to confer acquired resistance to EGFR therapy.^33,34^ Therefore, disclosure and inhibition of cellular protective feedback loops may prevent the emergence of therapeutic resistance. Previous studies have suggested resistance to anti-EGFR antibodies to be mainly mediated through the constitutive activation of EGFR downstream signalling cascades. Resistance development might be due to either genetic alterations in the RAS/RAF, PIK3CA/PTEN, or result from activation of growth receptors.^35^ Similarly, MAPK blockage has been demonstrated to reconstitute sensitivity to irreversible EGFR inhibition by TKI (tyrosine kinase inhibitor).^36^

This current study analysed the effects of EGFR inhibitor cetuximab on the activation of MAPK/ERK signalling, which is known to play a role in HNSCC tumorigenesis and treatment resistance development. We found the majority of HNSCC cell lines adapted the long-term cetuximab treatment by persistent or reactivation of ERK1/2 signalling as a mode of cellular defence mechanism. This adaptive response may facilitate the formation of tumour cells, which could be eliminated by a MEK1/2 inhibitor. These new results provide a proof-of-concept that up-regulation of ERK phosphorylation levels might contribute to cellular defence to cetuximab in HNSCC. Our data are in line with Yonesaka et al.^37^, who hypothesised that amplification of ErbB2 or upregulation of heregulin might lead to persistent ERK1/2 activation and cetuximab resistance. Furthermore, our experimental data revealed that PD-901, a MEK1/2 inhibitor, increases sensitivity of HNSCC cell lines to cetuximab and fractionated-IR, providing potential clinical benefit from a combination of MEK inhibitor with clinically available cetuximab treatment and radiotherapy. Adaptive resistance against EGFR inhibition in lung cancer cells was described by Ma et al.^38^ and was supposed to develop during initial therapy via feedback mechanisms that result in tumour cell survival and residual disease. The authors demonstrate that adaptive as well as acquired resistance to the EGFR inhibitor serlotinib, a TKI of EGFR, converged on MAPK activation.^38^

The rationale of combining the ErbB family inhibitor Afatinib with MEK inhibitor PD-901 has been recently demonstrated by Lin et al. in HNSCC cell lines. By combined treatment a compensatory upregulation of the MEK/ERK and the Akt/mTor cascade was abolished and proliferation and survival of platinum-resistant cancer cells was synergistically suppressed confirming the relevance of EGFR and MEK/ERK signalling in therapeutic response.^39^ It is known that in the case of pharmacological blockage of the EGFR receptor alternative signalling cascades/components become activated as a compensatory mechanism to escape receptor inhibition. This pathway activation is in most cases related to genomic alterations in downstream effectors (e.g., KRAS, NRAS, BRAF and PIK3CA) of the EGFR signalling pathway which converge on MAPK induction.^40^ Therefore, our findings are of clinical importance as currently various MEK inhibitors undergo clinical development (NCT03088176, NCT03972046, NCT02626000).

So far, adaptation of MAPK/ERK signalling in response to cetuximab has not been described for HNSCC. Zhang et al. reported re-activation of ERK after MEK inhibition in colorectal cancer cells which was overcome by cetuximab indicating that double MEK and EGFR blockade are crucial in epithelial cancer.^41^ We and others have shown that the MAPK/ERK axis plays a significant role in the development of radioresistance in HNSCC.^12,13,42^ IR mediates pathway activation, which leads to reduced radiosensitivity. However, these studies were based on treatment with a single dose. Healthy tissue and malignant tumours differ in their radiosensitivity. These radiobiological differences are used in dose fractionation. Healthy tissue can largely repair sublethal damage during radiation breaks, whereas recovery times for comparable repair procedures in malignant cells are often longer.^43^ Our intention was to adjust our in vitro setting to this schedule as precisely as possible. To realistically mimic the radiotherapeutic regimen applied to HNSCC patients, FaDu cells were treated with fractionated irradiation as a clinically relevant schedule. We observed a distinct activation of ERK1/2 phosphorylation, which was inhibited by PD-901. Post-radiogenic upregulation of ERK1/2 phosphorylation after fractionated IR was confirmed in ex vivo tumour cultures, however, with some variations, suggesting a context-dependent mode of regulation. These findings indicate that post-radiogenic activation of MAPK signalling most likely contributes to cellular radioprotective mechanism and can be radiosensitised by MEK inhibition in HNSCC cells. Integration of the ex vivo culture technology as a preclinical and experimental platform into further studies might be a useful strategy to stratify patient subgroups, which might benefit from novel treatment combinations.

Recently, elevated ERK1/2 phosphorylation was associated with shorter survival in patients with HNSCC.^44–46^ These studies provide evidence for a potential role of activated ERK1/2 as a predictive biomarker of clinical outcomes for HNSCC patients treated with RT, CT and/or cetuximab. Moreover, Kong et al. found an association between MEK/ERK upregulation and cisplatin resistance suggesting a role for the combined use of cisplatin with MEK inhibitors for patients with upregulated MAPK/ERK signalling in HNSCC.^45^

To determine, if ERK1/2 activation occurs in patient tumour samples and could potentially reduce the anti-tumour effect of cetuximab, we surveyed TMAs with newly diagnosed chemo-naive HNSCC, where the primary treatment included concurrent standard radiotherapy with single-agent cetuximab application. We discovered that the high-risk group of patients had higher levels of ERK1/2 and EGFR phosphorylation as compared to lower risk patients. As the number of samples for this cohort was small, we made use of publicly available data from the TCPA-HNSCC database and we were able to confirm the distribution into risk groups. Kaplan–Meier analysis of the TCPA cohort revealed that patients that were defined as high-risk group according to their protein expression profiles showed significantly worse survival. Thus, our analysis strongly suggests that activated ERK1/2 and activated EGFR are powerful predictors of poor prognosis in HNSCC patients. This is in line with results from previous findings stating that overexpression of EGFR tends to be associated with a shorter PFS in the cetuximab-treated group^47^ and our recent results on the correlation between ERK1/2 activation and poor prognosis in OPSCC.^46^

In summary, we hypothesise that adaptive phosphorylation of ERK might be a potential mechanism of cellular defence to cetuximab and radiotherapy in HNSCC, suggesting potential benefits of combining current therapeutic strategies with targeted therapy against MEK-ERK signalling. When evaluating a potential prognostic significance of the EGFR-MAPK axis, results from an in silico analysis of publicly available HNSCC cohorts and immunohistochemically assessed expression levels of activated ERK1/2 and EGFR revealed both markers to impact on clinical outcome in HNSCC.

## Supplementary information

SUPPLEMENTAL MATERIAL

## Data Availability

All data generated or analysed during this study are included in this publication. HNSCC datasets are available from TCGA/TCPA described in the methods session.
