# Supplementary material for: Adaptive ERK signalling activation in response to therapy and in silico prognostic evaluation of EGFR-MAPK in HNSCC
Source: Br J Cancer. 2020 May 19;123(2):288–97. doi: 10.1038/s41416-020-0892-9 (PMC7374086; doi:10.1038/s41416-020-0892-9)
Supplement: Supplementary file 1 — SUPPLEMENTAL MATERIAL [file 41416_2020_892_MOESM1_ESM.docx]

**Supplemental Table S1.** List of primary (**A**) and secondary antibodies (**B**) for Western blot analysis, immunofluorescence and immunohistochemical staining

| A | Cat. No. | Company | Application | Dilution |
| --- | --- | --- | --- | --- |
| phospho-ERK1/2 | CST9101 | Cell Signaling Technology | WB  IHC | 1:1,000  1:250 |
|  |  |  | IF | 1:250 |
| ERK1/2 | CST9102 | Cell Signaling Technology | WB  IHC | 1:1,000  1:100 |
| phospho-EGFR(Tyr1173) | CST4407 | Cell Signaling Technology | IHC | 1:50 |
| EGFR | CST4267 | Cell Signaling Technology | IHC | 1:50 |

*WB = Western blot, IHC = immunohistochemistry, IF = immunofluorescense*

| B | Cat. No | Company | Application | Dilution |
| --- | --- | --- | --- | --- |
| anti-mouse-HRP | SC-2005 | Santa Cruz | WB | 1:10,000 |
| anti-rabbit-HRP | SC-2317 | Santa Cruz | WB | 1:10,000 |
| anti-rabbit-Biotin | BA-1000 | Vector Laboratories | IHC | 1:200 |
| anti-Cy3 | 111-165-008 | Dianova | IF | 1:200 |
| ImmPress® goat |  | Vector | IHC |  |
| ImmPress® mouse |  | Vector | IHC |  |

*WB = Western blot, IHC = immunohistochemistry, IF = immunofluorescense*

**Supplemental Table S2**. Clinical features of HNSCC patients

| **Features** | **Category** | **N** | **%** |
| --- | --- | --- | --- |
| Age [years] | (median = 69.34) 22 | | |
| Gender | Male | 16 | 73 |
|  | Female | 6 | 27 |
| T status | T1-T2 | 6 | 27 |
|  | T3-T4 | 16 | 73 |
| N status | N0 | 8 | 36 |
|  | N+ | 14 | 64 |
| M status | M0 | 14 | 64 |
|  | M+ | 2 | 9 |
|  | Mx | 6 | 27 |
| Pathological grading | G1-2 | 8 | 36 |
|  | G3 | 2 | 9 |
|  | missing | 12 | 55 |
| Alcohol | no/former | 8 | 36 |
|  | current  missing | 7  7 | 32  32 |
| Tobacco | no/former | 7 | 32 |
|  | current  missing | 10  5 | 45  23 |

**Supplemental** **Figure S1.** PD-901 treatment sensitizes HNSCC cell lines to fractionated IR and cetuximab treatment. Representative staining of a CFA with control (DMSO), cetuximab (5μg/ml) and cetuximab plus PD-901 (0.1μM) with or without fractionated-IR in FaDu (a) and Cal27 (b) cells. (c) The graph represents the relative survival fraction of HNSCC cell lines after treatment with cetuximab or (and) PD-901 with or without fractionated-IR. DMSO-treated control cells were normalized to one and bars represent mean values ± SEM of three independent experiments.

**Supplemental** **Figure S2.** Prognostic analysis of EGFR-MEK-ERK gene signature in two independent HNSCC cohorts. Overall survival of risk groups was evaluated by Kaplan–Meier survival plot and Log-Rank test in a cohort of 86 oral cancer patients from The University of Texas M.D. Anderson Cancer Center, US (a) and a cohort of 89 HNSCC patients from Institut National de la Sante et de la Recherche Medicale (INSERM), France (b). Total number of patients at risk were displayed at indicated time points.


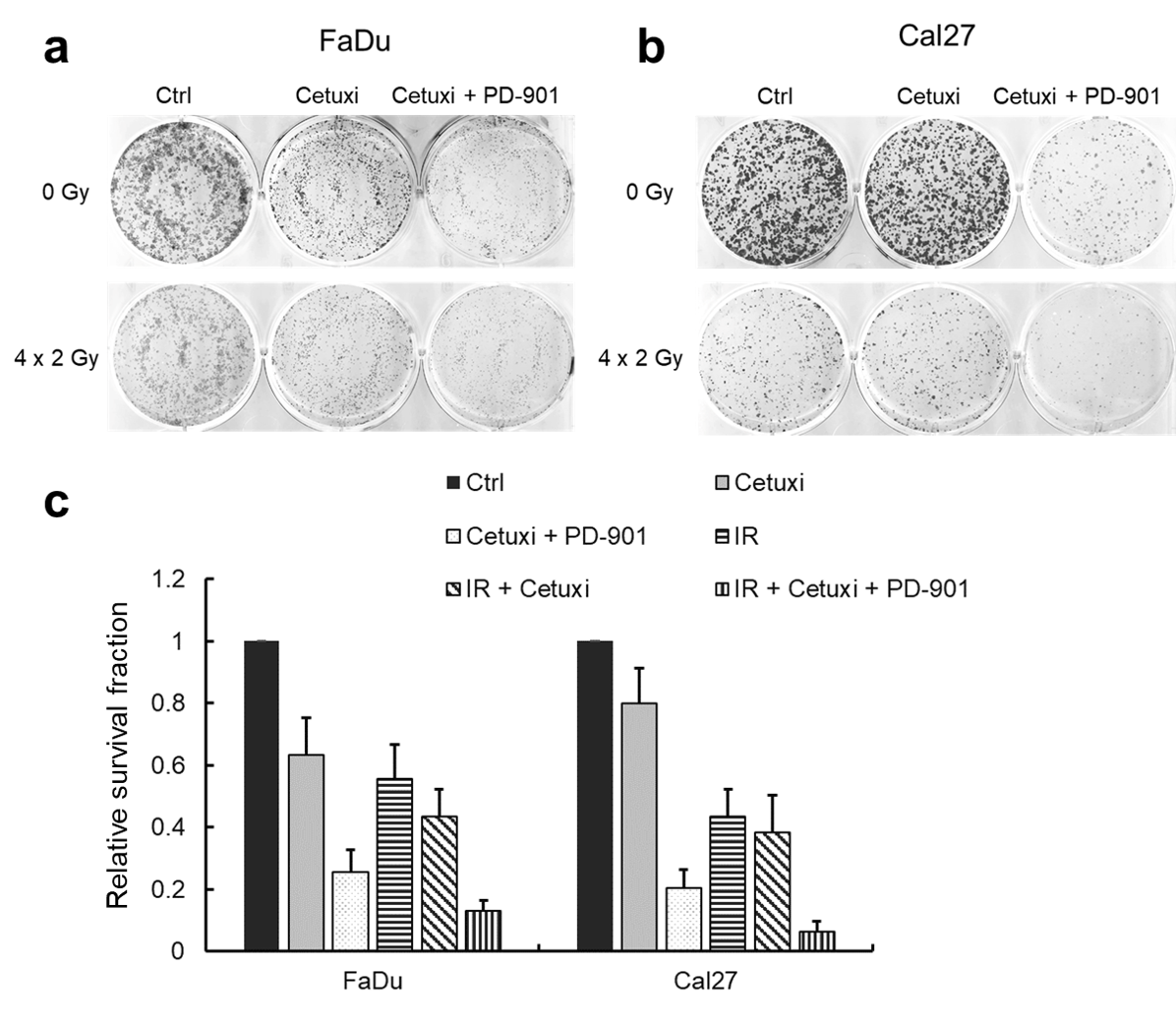


***Supplemental*** ***Figure S1***


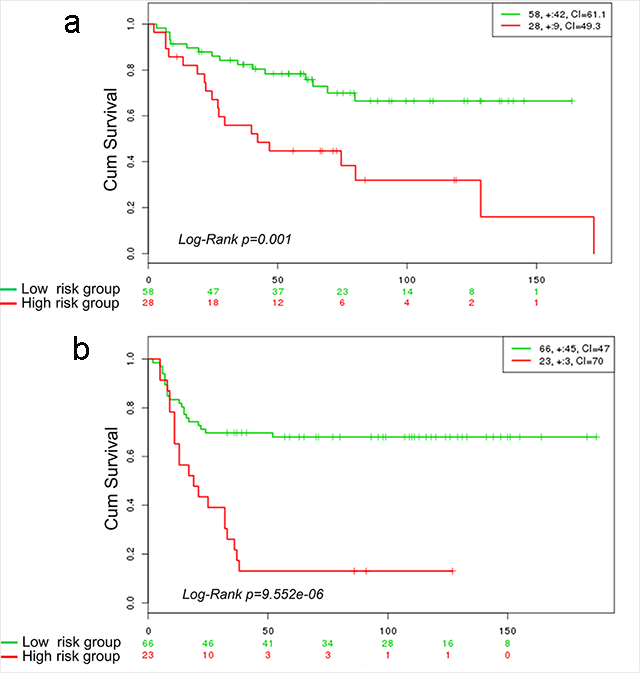


***Supplemental*** ***Figure S2***
